# Supplementary material for: Changes in human peripheral blood mononuclear cell (HPBMC) populations and T-cell subsets associated with arsenic and polycyclic aromatic hydrocarbon exposures in a Bangladesh cohort
Source: PLoS One. 2019 Jul 31;14(7):e0220451. doi: 10.1371/journal.pone.0220451 (PMC6668812; doi:10.1371/journal.pone.0220451)
Supplement: S1 Fig — Flow chart indicating the gating strategy for CSM. This flow chart has been modified from the original by Lauer et al. [34] to include activated B cells. (PDF) [file pone.0220451.s001.pdf]

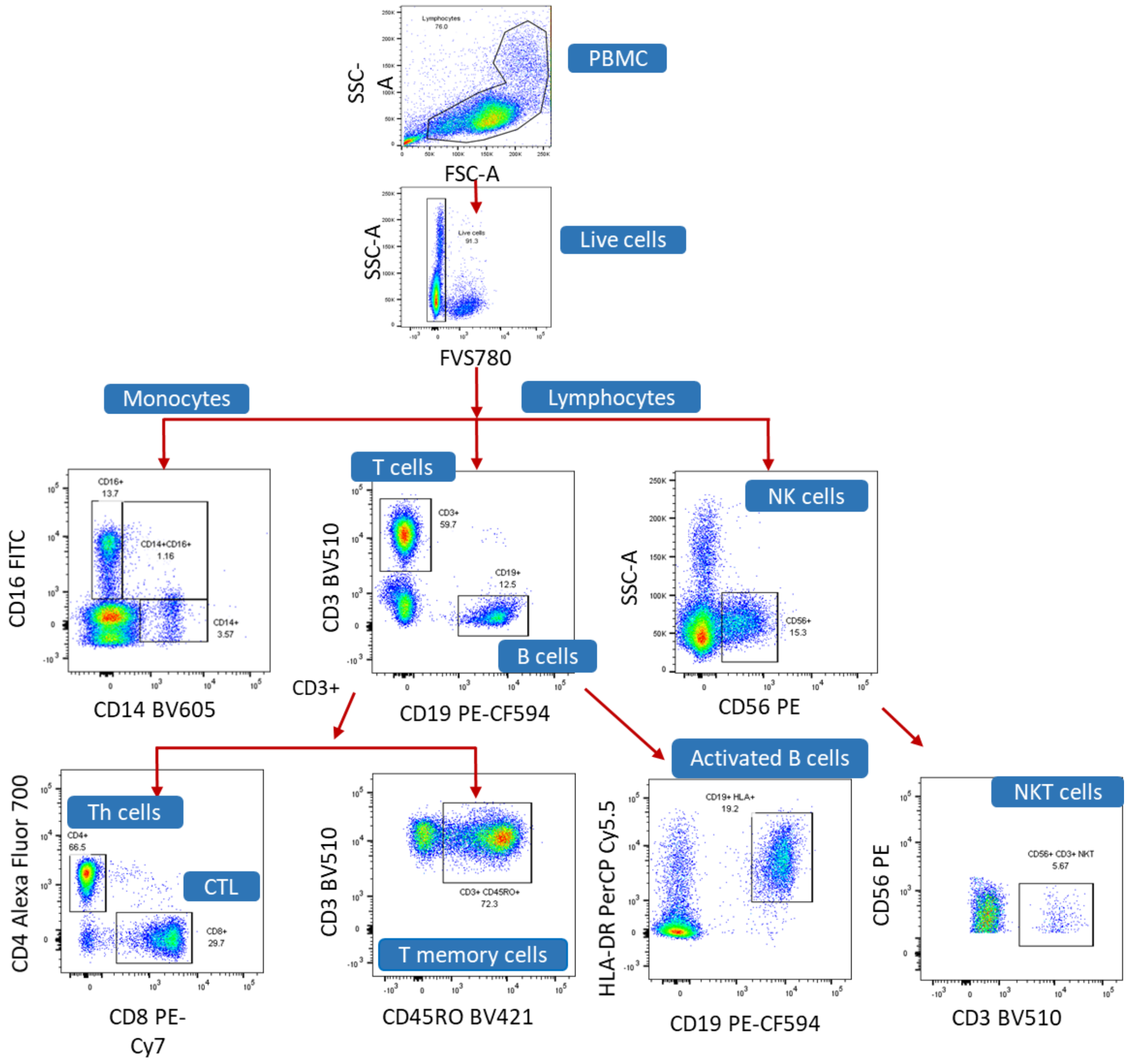

Fig S1: Gating Strategy for Cell Surface Markers.

Flow chart indicating the gating strategy for CSM. This flow chart has been modified from the original by Lauer et al. [34] to include activated B cells.
