# Supplementary material for: Changes in human peripheral blood mononuclear cell (HPBMC) populations and T-cell subsets associated with arsenic and polycyclic aromatic hydrocarbon exposures in a Bangladesh cohort
Source: PLoS One. 2019 Jul 31;14(7):e0220451. doi: 10.1371/journal.pone.0220451 (PMC6668812; doi:10.1371/journal.pone.0220451)
Supplement: S2 Fig — Flow chart indicating the gating strategy for ICS by Lauer et al. [35]. (PDF) [file pone.0220451.s002.pdf]

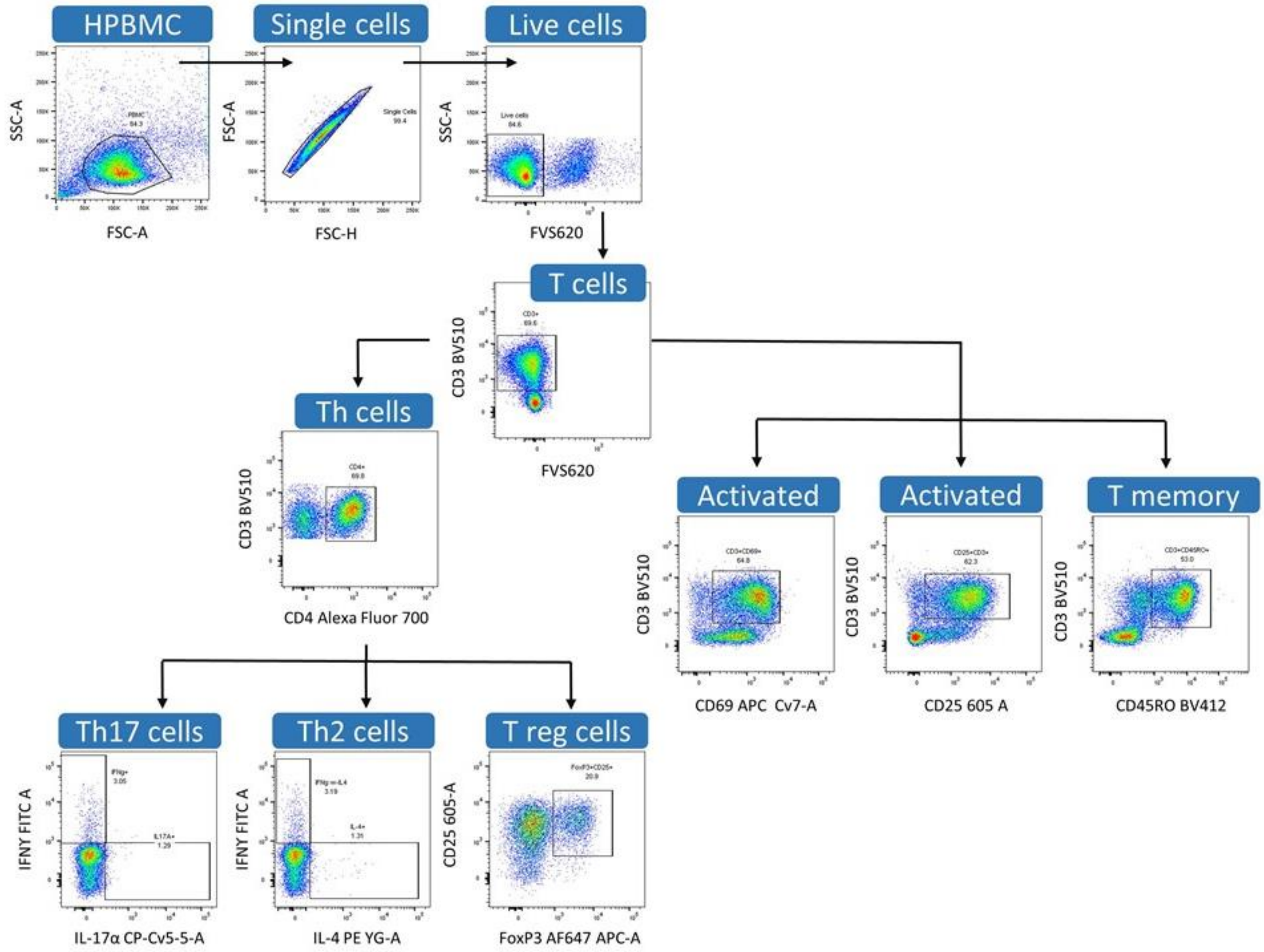

Fig S2: Gating Strategy for Intracellular Markers

Flow chart indicating the gating strategy for ICS by Lauer et al. [35].
