# Supplementary material for: Changes in human peripheral blood mononuclear cell (HPBMC) populations and T-cell subsets associated with arsenic and polycyclic aromatic hydrocarbon exposures in a Bangladesh cohort
Source: PLoS One. 2019 Jul 31;14(7):e0220451. doi: 10.1371/journal.pone.0220451 (PMC6668812; doi:10.1371/journal.pone.0220451)
Supplement: S1 Table — (PDF) [file pone.0220451.s003.pdf]

S1 Table. Cell surface marker (CSM) antibodies for flow cytometry.

Laser	Filter	Fluorochrome	Specificity	Clone	Cat. No.	Volume (µl)
Blue 488 nm	530/30	FITC	CD16	3G8	555406	20
	695/40	PerCP-Cy™5.5	HLA-DR	G46-6	560652	5
Yellow-Green 561 nm	582/15	PE	CD56	B159	555516	20
	610/20	PE-CF594	CD19	HIB-19	562294	5
	780/60	PE-Cy™7	CD8	RPA-T8	557746	5
Red 640 nm	670/14	Alexa Fluor 647	CD127	HIL-7R-M21	558598	20
	730/45	Alexa Fluor 700	CD4	RPA-T4	557922	5
	780/60	APC-Cy™7	FVS780		565388	1
Violet 405 nm	450/50	BV421	CD45RO	UCHL1	562641	5
	525/50	BV510	CD3	UCHT1	563109	5
	605/12	BV605	CD14	M5E2	564054	5

Note: To ensure limited inter-assay variability for batch analysis the antibody volumes are manufacturer's recommendations.
