# Supplementary material for: Changes in human peripheral blood mononuclear cell (HPBMC) populations and T-cell subsets associated with arsenic and polycyclic aromatic hydrocarbon exposures in a Bangladesh cohort
Source: PLoS One. 2019 Jul 31;14(7):e0220451. doi: 10.1371/journal.pone.0220451 (PMC6668812; doi:10.1371/journal.pone.0220451)
Supplement: S2 Table — (PDF) [file pone.0220451.s004.pdf]

S2 Table. Intracellular marker (ICM) antibodies for flow cytometry.

Laser	Filter	Fluorochrome	Specificity	Clone	Cat. No.	Volume (µl)
Blue 488 nm	530/30	FITC	IFNγ	B27	552887	20
	695/40	PerCP-Cy™5.5	IL-17A	N49-653	560799	5
Yellow-Green 561 nm	582/15	PE	IL-4	8D4-8	559333	20
	610/20	PE-CF594	FVS620*		564996	1
	780/60	PE-Cy™7	CD127*	HIL-7R-M21	560822	5
Red 640 nm	670/14	Alexa Fluor 647	Foxp3	259D/C7	560822	20
	730/45	Alexa Fluor 700	CD4*	RPA-T4	557922	5
	780/60	APC-Cy™7	CD69*	FN50	557756	5
Violet 405 nm	450/50	BV421	CD45RO*	UCHL1	562641	5
	525/50	BV510	CD3*	UCHT1	563109	5
	605/12	BV605	CD25*	2A3	562660	5

Note: To ensure limited inter-assay variability for batch analysis the antibody volumes are manufacturer’s recommendations.
*indicates CSM
