# Supplementary material for: Changes in human peripheral blood mononuclear cell (HPBMC) populations and T-cell subsets associated with arsenic and polycyclic aromatic hydrocarbon exposures in a Bangladesh cohort
Source: PLoS One. 2019 Jul 31;14(7):e0220451. doi: 10.1371/journal.pone.0220451 (PMC6668812; doi:10.1371/journal.pone.0220451)
Supplement: S3 Table — (PDF) [file pone.0220451.s005.pdf]

S3 Table. Cell Surface Markers (CSM)

Designation	Marker
T cell	CD3+
Th	CD4+CD8-
CTL	CD8+CD4-
Tmem	CD3+CD45RO+
MonoClass	CD14+CD16-
NonMono	CD14+CD16+
Bcell	CD19+
ActB	CD19+HLA-DR+
NK	CD56+CD3-
NKT	CD56+CD3+
IL7Ra	CD127+
