# Supplementary material for: Changes in human peripheral blood mononuclear cell (HPBMC) populations and T-cell subsets associated with arsenic and polycyclic aromatic hydrocarbon exposures in a Bangladesh cohort
Source: PLoS One. 2019 Jul 31;14(7):e0220451. doi: 10.1371/journal.pone.0220451 (PMC6668812; doi:10.1371/journal.pone.0220451)
Supplement: S4 Table — (PDF) [file pone.0220451.s006.pdf]

S4 Table. Intracellular Staining (ICS)

Designation	Marker
CD4	CD4+
Treg	CD3+CD4+Foxp3+CD25+
Th1	CD3+CD4+IFNg+
Th2	CD3+CD4+IL4+
Th17A	CD3+CD4+IL17A+
StimCD4	CD3+CD4+CD69+CD25+
StimCD3	CD3+CD69+CD25+
StimLive	Live cells CD69+CD25+
